# Interkingdom Endodontic Biofilm Supernatant Induces a Biphasic Inflammatory and Metabolic Transcriptional Response in Dental Pulp Stem Cells In Vitro

**DOI:** 10.1002/cre2.70417

**Published:** 2026-07-19

**Authors:** Saeed S. Alqahtani, Sumaya Abusrewil, Othman Baradwan, Om Alkhir Alshanta, Ryan Kean, William McLean, Jason L. Brown, Christopher J. Nile

**Affiliations:** ^1^ Department of Restorative Dental Sciences, College of Dentistry Jouf University Al‐Jouf Saudi Arabia; ^2^ Department of Operative Dentistry and Endodontics, School of Dentistry University of Tripoli Tripoli Libya; ^3^ Oral Sciences Research Group, Glasgow Dental School University of Glasgow Glasgow UK; ^4^ Department of Biological and Biomedical Sciences, School for Health and Life Sciences Glasgow Caledonian University Glasgow UK; ^5^ Faculty of Medical Sciences, School of Dental Sciences Newcastle University Newcastle upon Tyne UK

**Keywords:** dental pulp stem cells, endodontic biofilm, inflammatory response, regeneration, vital pulp therapy

## Abstract

**Objectives:**

To investigate how soluble byproducts derived from a four‐species endodontic biofilm model impact the viability, transcriptomic profile, and inflammatory response of human dental pulp stem cells (DPSCs).

**Methods:**

A sterile‐filtered supernatant was extracted from an established interkingdom endodontic biofilm model comprising *Streptococcus gordonii*, *Fusobacterium nucleatum*, *Porphyromonas gingivalis,* and *Candida albicans*. DPSCs were exposed to the microbial biofilm supernatant (BSN) for 4 and 24 h. Cellular responses were evaluated via MTT, CCK‐8, LDH assays, and Annexin V/PI staining. Transcriptomic sequencing was performed to assess gene expression dynamics, with GO and KEGG pathway enrichment analyses. IL6 and IL8 expression was validated by qPCR and ELISA. Data were analyzed using t‐tests/ANOVA and RNA‐seq differential expression using DESeq. 2 with FDR adjustment.

**Results:**

BSN significantly suppressed DPSC metabolic activity without inducing apoptosis or necrosis. RNA‐seq revealed 723 significantly differentially expressed genes at 4 h and 1667 at 24 h. Early responses were dominated by upregulation of inflammatory mediators, with enrichment of TNF, NF‐κB, and JAK‐STAT signaling pathways. At 24 h, the expression profile shifted toward redox regulation and metabolic suppression, including downregulation of glycolytic and purine metabolism pathways. IL6 and IL8 expression was markedly increased at both transcript and protein levels.

**Conclusions:**

Soluble factors produced by a biofilm model representative of deep caries and carious pulp exposures induce a time‐dependent transcriptional response in DPSCs. This response is characterized by a biphasic pattern of early immune activation followed by later transcriptional metabolic adaptation. These findings highlight the capacity of soluble biofilm‐derived products associated with deep caries to modulate DPSC immune–metabolic signaling. They further emphasize the importance of vital pulp therapy strategies that not only target microorganisms but also account for their secreted byproducts.

## 1 Introduction

Dental pulp is a highly specialized connective tissue that is susceptible to inflammation triggered by microbial invasion, commonly through dental caries or trauma (Pohl et al. 2024). Microbial ingress into the pulp and root canal system initiates pulpitis, which may progress to necrosis and apical periodontitis. As pulpitis severity and clinical presentation are variable, contemporary management increasingly emphasizes preserving pulp vitality where possible. Clinically, pulpitis is typically classified as reversible or irreversible. The distinction between these two states is somewhat subjective and does not acknowledge the underlying biological status. The degree of inflammation is central to treatment planning, yet the classification of reversible or irreversible pulpitis without direct inspection of the pulp risks inappropriate treatment choices. Moreover, conditions once considered irreversible may now be recognized as potentially reversible. A new classification has therefore been proposed and subsequently revised due to a deeper understanding of pulp biology (American Association of Endodontists 2022; Karrar et al. 2025).

Vital pulp therapy (VPT) has emerged as a biologically informed alternative to root canal treatment in selected cases of inflamed but still vital pulp (El karim et al. 2021; Zhang and Yelick 2010). This shift reflects evidence that resident pulp cells can contribute to defense, healing, and regeneration, and that even severely inflamed pulp may retain reparative capacity (Colombo et al. 2014; Kahler et al. 2023). Pulp inflammation is characterized by upregulation of cytokines, chemokines, and complement proteins that amplify immune responses and recruit inflammatory cells (Zanini et al. 2017; Hirsch et al. 2017; Hahn et al. 1989). While protective initially, sustained inflammation can lead to collateral tissue damage, including extracellular matrix breakdown and loss of pulp vitality (Cooper et al. 2017). Improving VPT outcomes, therefore, requires strategies that control inflammation while preserving regenerative cell function. VPT targets inflamed, yet still vital pulp, where dental pulp stem cells (DPSCs) remain present and functionally relevant (Scelza et al. 2021; Saoud et al. 2016). Therefore, for successful VPT, medicaments that can modulate the excessive inflammatory response of the pulp tissue while preserving the regenerative potential of DPSCs may be imperative for favorable clinical outcomes. These processes are strongly influenced by inflammatory and metabolic cues within the pulp microenvironment.

DPSCs contribute to innate immune signaling and tissue repair during infection. However, most studies investigating their responses to microorganisms have used reductionist stimuli (e.g., single‐species or individual‐specific microbial antigens such as LPS). Previous studies have demonstrated that Toll‐like receptor (TLR) and subsequent downstream nuclear factor kappa‐light‐chain‐enhancer of activated B cells (NF‐κB) activation causes upregulated expression of inflammatory mediators, such as IL‐6 and IL‐8, often alongside impaired odontogenic differentiation (Kim et al. 2023). However, how DPSCs respond to soluble factors released by clinically relevant mixed species polymicrobial biofilms remains less well defined.

Soluble factors released by microbial biofilms can impair mesenchymal stem cell (MSCs) viability, migration, and differentiation (Ward et al. 2015). In endodontics, biofilms can persist despite chemomechanical procedures (Nair 2006), and residual microbial products can remain within dentine and influence host cells after disinfection (Vishwanat et al. 2017; Diogenes and Hargreaves 2017; Brizuela et al. 2022). Deep dentinal caries and carious pulp exposures harbor polymicrobial communities that include streptococci, Fusobacterium, Porphyromonas, and Candida species (Rôças et al. 2015, 2016; Neves et al. 2017; Kuzmanović Radman et al. 2019; Kirilova et al. 2019). *Fusobacterium nucleatum* contributes to polymicrobial biofilm structure and inflammatory signaling, while *Candida albicans* can persist in dentinal niches and engage in inter‐kingdom interactions that modulate host responses (Lima et al. 2011; Yoo et al. 2020; Du et al. 2022). Collectively, these communities release diffusible products, including soluble microbial metabolites, virulence factors, and other secreted proteins that can traverse dentine and influence pulp cell behavior without direct microbial invasion (Rôças et al. 2015; Yoo et al. 2020; Du et al. 2022). However, the effects of these polymicrobial, interkingdom biofilm‐derived soluble factors on DPSC remain incompletely understood.

The aim of this study was to investigate how soluble factors derived from a four‐species interkingdom endodontic biofilm model, which includes *S. gordonii*, *P. gingivalis*, *F. nucleatum*, and *C. albicans*, affect DPSC viability, gene expression, and inflammatory signaling. Using transcriptomic profiling, we identify a biphasic gene expression pattern characterized by early immune activation followed by later transcriptional metabolic adaptation, with relevance to pulp vitality in deep caries and carious exposure.

## 2 Materials and Methods

The materials and methods have been written according to Preferred Reporting Items for Laboratory studies in Endodontology (PRILE) 2021 guidelines.

### 2.1 Culture of Primary Human Dental Pulp Stem Cells

Primary human dental pulp stem cells (DPSCs) (Lonza Inc., UK) were cultured at 37°C in 5% CO_2_ in Knock‐out Dulbecco's Modified Eagle's Medium (DMEM‐KO) (Gibco, UK) supplemented with 10% Fetal Bovine Serum, 1% L‐Glutamine (Sigma, UK‎), and 1% Penicillin‐Streptomycin (Gibco, UK). According to the supplier, these DPSCs were derived from a single healthy donor and were characterized prior to distribution based on established mesenchymal stem cell criteria, including surface marker expression and multipotent differentiation capacity. Media was changed every 3 days. When approximately 70%–80% confluent, cells were passaged by detaching with 0.025% trypsin–EDTA (Gibco, UK). All DPSCs used in this study were between passages 4 and 6.

### 2.2 Polymicrobial Biofilm Formation

An established, validated interkingdom endodontic biofilm model, containing *Streptococcus gordonii* (ATCC 35105), *Porphyromonas gingivalis* (ATCC 33277), *Fusobacterium nucleatum* (ATCC 10953), and *Candida albicans* SC5314 (ATCC MYA‐2876) was used throughout this study (Abusrewil et al. 2020). *Streptococcus gordonii* was grown on Columbia blood agar supplemented with 5% horse blood at 37°C in 5% CO_2_ for 24 h. *P. gingivalis* and *F. nucleatum* were cultured on fastidious anaerobic agar plates, containing 5% defibrinated horse blood and maintained at 37°C in an anaerobic incubator (Don Whitley Scientific Limited, UK) with an atmosphere of 85% N_2_, 10% CO_2,_ and 5% H_2_ for 24–48 h. *Candida albicans* was cultured on Sabouraud dextrose agar and incubated aerobically for 24–48 h at 30°C. Cultures of *C. albicans* and bacterial species, standardized at 1 × 10^8^ CFU/mL, were diluted to 1 × 10^6^ CFU/mL and 1 × 10^7^ CFU/mL in the culture broth, respectively. The broth for the multispecies biofilm consisted of Roswell Park Memorial Institute‐1640 (RPMI) with Todd Hewitt Broth (THB) supplemented with 0.01 mg/mL hemin and 2 μg/mL menadione. The four‐species biofilm was grown in T‐25 cell culture flasks (Corning, NY, USA) for 24 h in 5% CO_2_ at 37°C. Following incubation, the supernatant was centrifuged at 4000 rpm for 5 min before filtering with a sterile 0.2 μm syringe filter (Sartorius Minisart, Fisher Scientific, UK). All procedures were performed under aseptic conditions in a Class II biosafety cabinet using sterile plastics and media. Work surfaces and instruments were disinfected before and after each session. The preparation of BSN was intended to preserve biologically active soluble factors released by the multispecies biofilm for functional cellular assays, rather than to enable detailed biochemical profiling.

### 2.3 Microbial Biofilm Supernatant Co‐Culture With Human Dental Pulp Stem Cells

DPSCs at 70%–80% confluency were exposed to sterile‐filtered supernatant derived from the four‐species microbial biofilm (BSN) mixed with complete DMEM‐KO medium at the following dilutions: 1:1, 1:2, 1:3, and 1:4 (v/v). BSN dilutions were prepared in complete culture medium to preserve physiological buffering and minimize non‐specific stress responses. Untreated cells maintained in standard DPSC medium served as negative controls. After 24 h of incubation, cell viability and cytotoxicity were assessed using MTT, LDH release, and CCK8 assays across all tested dilutions. Experimental categories were pre‐specified as BSN dilutions (1:1, 1:2, 1:3, 1:4) and a negative control (untreated medium); control nomenclature is consistent across assays and figures.

### 2.4 MTT Assay

To evaluate the effect of BSN on DPSC metabolic activity, a 3‐(4,5‐dimethylthiazol‐2‐yl)‐2,5‐diphenyltetrazolium bromide (MTT) assay was performed. DPSCs were seeded in 96‐well plates at a density of 1 × 10^4^ cells/well in complete DMEM‐KO medium and allowed to adhere for 24 h at 37°C in 5% CO_2_. Cells were then treated with a 1:1 mixture of BSN and culture medium. Two control groups were included: untreated cells cultured in standard medium (positive control), and cells cultured in serum‐free DMEM‐KO (no FBS; negative control). After 24 h incubation, the culture media were removed and replaced with 100 µL of MTT solution (0.5 mg/mL in serum‐free DMEM‐KO; Sigma‐Aldrich, UK). Plates were incubated for 4 h at 37°C in 5% CO_2_ to allow for formazan crystal formation. The MTT solution was then removed, and 100 µL of dimethyl sulfoxide (DMSO; Sigma‐Aldrich, UK) was added to each well to solubilize the crystals. Plates were incubated for a further 1 h at 37°C. Absorbance was measured using a FLUOstar Omega microplate reader (BMG Labtech, Germany) at 545 nm with background correction at 650 nm. Metabolic activity was calculated relative to the untreated control.

### 2.5 LDH Assay

To evaluate plasma membrane damage in DPSCs following exposure to BSN, lactate dehydrogenase (LDH) release was measured using the Pierce LDH Cytotoxicity Assay Kit (Thermo Fisher Scientific, UK) according to the manufacturer's instructions. DPSCs were seeded in 96‐well plates at a density of 1 × 10^4^ cells/well in complete DMEM‐KO medium and allowed to adhere for 24 h. Cells were then treated with a 1:1 mixture of BSN and culture medium for 24 h. Following treatment, 20 µL of culture supernatant was transferred to a new 96‐well plate and mixed with 20 µL of LDH reaction mixture. Samples were incubated in the dark for 30 min at room temperature, and the reaction was stopped by adding 20 µL of stop solution. Absorbance was measured at 490 nm with background correction at 680 nm using a FLUOstar Omega microplate reader (BMG Labtech, Germany). Three control groups were included: (i) cells treated with lysis buffer (positive control), (ii) untreated cells in standard culture medium (baseline control), and (iii) untreated cells cultured in serum‐free DMEM‐KO (negative control). Percentage cytotoxicity was calculated according to the manufacturer's instructions.

### 2.6 CCK‐8 Assay

To assess the effect of BSN on the viability of metabolically active DPSCs, a Cell Counting Kit‐8 (CCK‐8; Dojindo Molecular Technologies, Japan) assay was performed according to the manufacturer's protocol. DPSCs were seeded in 96‐well plates at a density of 1 × 10^4^ cells/well in complete DMEM‐KO medium and allowed to adhere for 24 h. Cells were then treated with a 1:1 mixture of BSN and culture medium for 24 h. Two control groups were included: (i) untreated cells in standard culture medium (positive control) and (ii) cells cultured in serum‐free DMEM‐KO (negative control). After the 24‐h incubation, 10 µL of CCK‐8 solution was added to each well, and the plate was incubated for 4 h at 37°C in 5% CO_2_. Absorbance was measured at 450 nm using a FLUOstar Omega microplate reader (BMG Labtech, Germany). Cell viability was calculated relative to the untreated control group.

### 2.7 Annexin V/PI Staining

To assess whether BSN exposure induced apoptosis or necrosis in DPSCs, an Annexin V–fluorescein isothiocyanate (FITC)/propidium iodide (PI) detection kit (Abcam, UK) was used according to the manufacturer's protocol. DPSCs were seeded in 24‐well plates at a density of 5 × 10^4^ cells/well in complete DMEM‐KO medium and allowed to adhere for 24 h. Cells were then treated with a 1:1 mixture of BSN and culture medium for 24 h. Positive controls for apoptosis and necrosis were generated by treating cells with 30% and 70% methanol, respectively, as methanol is commonly used to induce cytotoxicity and apoptotic markers in cell death studies (Spyridopoulos et al. 2001; Nguyen et al. 2020). After treatment, cells were sequentially incubated with 5 µL PI (50 µg/mL) and 5 µL Annexin V‐FITC (10 µg/mL) in binding buffer, in the dark, at room temperature. Following staining, cells were fixed with 2% paraformaldehyde in PBS for 15 min, washed with PBS, and mounted using DAPI‐containing mounting medium. Fluorescence imaging was performed using an EVOS FL digital inverted microscope (Thermo Fisher Scientific, UK) equipped with a monochrome camera and 40× phase contrast objective. Two‐channel overlay images were generated using the microscope's built‐in imaging software.

### 2.8 RNA Sequencing

DPSCs (5 × 10^5^ cells) were treated with a 1:1 mixture of BSN and culture medium for 4 and 24 h. Following treatment, total RNA was extracted using the RNeasy Mini Kit with on‐column DNase I digestion (Qiagen Ltd., UK) according to the manufacturer's instructions. RNA concentration and integrity were assessed using a Bioanalyzer 2100 system (Agilent Technologies, CA, USA). Only samples with a minimum RNA integrity number (RIN) of 7.0 and a concentration ≥ 20 ng/µL were processed for sequencing. RNA samples were submitted to Novogene Co. Ltd. (Cambridge, UK) for library preparation and RNA sequencing. Bioinformatic analysis was performed using in‐house Perl scripts according to the workflow described in Figure S1A.

### 2.9 Quantitative PCR

DPSCs were seeded at 1 × 10^5^ cells per well in 24‐well plates and incubated overnight at 37°C in a humidified atmosphere with 5% CO_2_. Cells were then treated with a 1:1 mixture of BSN and culture medium for 24 h. Total RNA was extracted using the RNeasy Mini Kit with on‐column DNase I treatment (Qiagen Ltd., UK), following the manufacturer's instructions. cDNA was synthesized from 1 µg of total RNA using the High‐Capacity cDNA Reverse Transcription Kit (Applied Biosystems, UK). Quantitative PCR (qPCR) was performed using a StepOnePlus Real‐Time PCR System (Applied Biosystems, UK) with SYBR Green Master Mix under the following cycling conditions: polymerase activation at 95°C for 2 min, followed by 40 cycles of 95°C for 5 s and 60°C for 30 s. Each reaction was run in duplicate. Expression levels of *IL8* and *IL6* genes were normalized to the housekeeping gene *GAPDH*, and relative gene expression was calculated using the e‐ΔCT method (Riedel et al. 2014). Primer sequences for *IL8*, *IL6,* and *GAPDH* are shown in Table S1.

### 2.10 Enzyme‐Linked Immunosorbent Assays

To quantify secreted IL8 and IL6 protein levels in response to BSN exposure, an IL8 and IL6‐specific enzyme‐linked immunosorbent assay (ELISA) was performed using commercially available kits (Invitrogen, UK), following the manufacturer's instructions. DPSCs were treated with a 1:1 mixture of BSN and complete culture medium for 24 h. After incubation, culture supernatants were collected, centrifuged to remove debris, and stored at –80°C until analysis. Samples and standards were added to the ELISA plate and incubated according to the kit protocol. After the final substrate reaction step, absorbance was measured at 450 nm using a FLUOstar Omega microplate reader (BMG Labtech, Germany). Cytokine concentrations were determined using known IL6 or IL8 concentrations provided by the kit manufacturer.

### 2.11 Statistical Analysis

Statistical analysis was performed with GraphPad Prism for macOS (GraphPad Software, version 10.1.1 Inc., La Jolla, CA, USA). Normal distribution of the investigated samples was assessed using the Shapiro–Wilk normality test. Analysis of two conditions was done with an unpaired *t*‐test followed by a Mann–Whitney test. Analysis of more than two conditions were done by one‐way ANOVA with Dunnett's multiple comparison or Kruskal–Wallis and Dunn's tests. The data are presented as mean ± standard error of the mean (SEM), with a difference of *p* < 0.05 considered statistically significant. Differential gene expression analysis of the RNA sequencing data is presented as *p*‐adjusted values (*p*adj) using Benjamini and Hochberg's approach for controlling the false discovery rate. Genes with a *p*adj <=0.05 found by DESeq. 2 were considered statistically significant.

### 2.12 Randomization, Allocation, and Blinding

This was an in vitro cell culture study with predefined plate maps and identical processing across groups. Randomization and allocation concealment were not applicable. Outcome assessment relied on instrument‐generated readouts (absorbance/fluorescence) and standardized acquisition settings; examiner blinding was not applicable.

### 2.13 Sample Size and Replicates

Each experiment was three independent biological repeats with technical replication as specified per assay (e.g., 96‐well formats at 1 × 10^4^ cells/well). For transcriptomic analyses, RNA sequencing was performed using four independent biological replicates per condition, with no technical replicates. This replication level is standard for exploratory in vitro studies of DPSC responses and, combined with multiple orthogonal assays (MTT, CCK‐8, LDH, Annexin V/PI), provides adequate precision for detecting biologically relevant effects.

### 2.14 Ethical Approval

Ethical approval was not required. Human dental pulp stem cells (DPSCs) were commercially sourced from Lonza (UK) and used in vitro; no human participants or animals were directly involved.

## 3 Results

### 3.1 Supernatant From a Four Species Interkingdom Endodontic Biofilm Reduces Dental Pulp Stem Cell Metabolic Activity Without Causing Cell Death

To determine whether exposure to supernatant from a four‐species interkingdom endodontic biofilm (BSN) affects DPSC viability, metabolic activity, and membrane integrity; MTT, CCK‐8, and LDH assays were initially employed.

**Figure 1:**
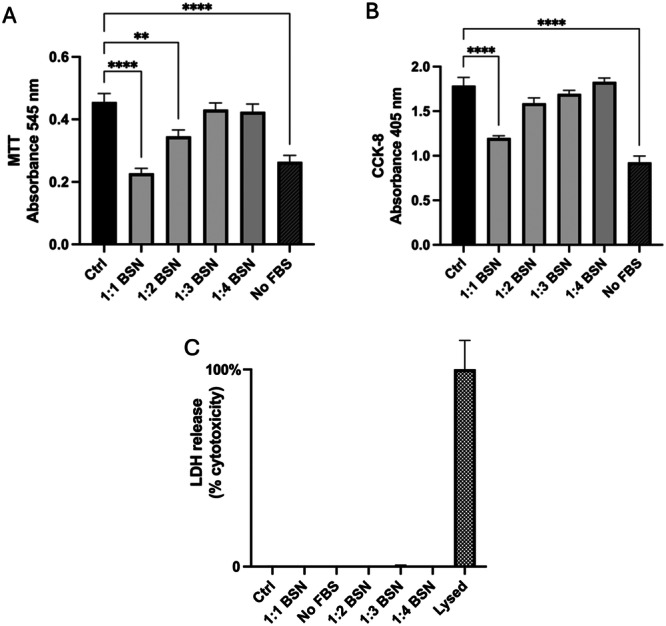
Biofilm supernatant (BSN) suppresses DPSC metabolic activity without inducing cytotoxicity. (A) MTT assay showing reduced metabolic activity in DPSCs after 24 h exposure to 1:1 and 1:2 BSN dilutions. (B) CCK‐8 assay confirming decreased metabolic activity following 1:1 BSN treatment. DPSCs in complete culture media (Ctrl) and cells in serum‐free (No FBS) media served as controls. (C) LDH‐based cytotoxicity analysis showing no increase in membrane damage after exposure of DPSCs to any dilution of BSN, compared to lysed cells. Ctrl = untreated DPSCs in complete medium; BSN = 1:1 dilution of biofilm supernatant in complete medium; No FBS = serum‐free negative control; Lysed = positive control for cytotoxicity. Data are presented as mean ± SEM from three independent experiments. *p < 0.05, **p < 0.01, ****p < 0.0001.

MTT analysis revealed a significant reduction in metabolic activity following 24‐h exposure to 1:1 BSN (*p* < 0.0001) and 1:2 BSN (*p* < 0.01) compared to the untreated control (Figure 1A). CCK‐8 analysis confirmed a significant reduction in metabolic activity in 1:1 BSN‐treated cells relative to the untreated control (*p* < 0.0001) (Figure 1B). To determine whether this reduction was due to cytotoxicity, LDH release was measured. LDH analysis confirmed no cytotoxic effects on DPSCs of any of the BSN dilutions after 24 h treatment (Figure 1C). Together, these findings suggest that BSN suppresses DPSC proliferation without inducing cell necrosis. Based on these preliminary findings, the 1:1 dilution of BSN was selected for all subsequent experiments as the highest exposure condition that reproducibly altered DPSC metabolic activity without inducing cytotoxicity or cell death. Between‐group effects are summarized with mean differences and 95% CIs in Table S2; LDH showed no increase versus control. No samples were lost or excluded from analysis at any stage.

**Figure 2:**
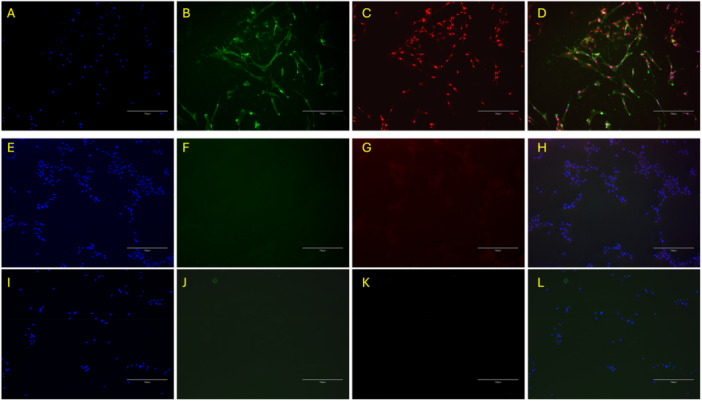
Annexin V/PI staining shows the absence of apoptosis or necrosis in DPSCs treated with biofilm supernatant (BSN). (A) DAPI nuclear staining of DPSCs exposed to 30% methanol (apoptosis control). (B) Annexin V‐FITC staining (green) shows apoptotic cells. (C) PI staining (red) confirms necrosis in cells exposed to 70% methanol (necrosis control). (D) Merged image of A–C. (E) DAPI nuclear staining of untreated DPSCs. (F) No Annexin V signal. (G) No PI signal. (H) Merged image of E–G. (I) DAPI nuclear staining of DPSCs treated with 1:1 BSN for 24 h. (J) No Annexin V signal. (K) No PI signal. (L) Merged image of I–K. Images are representative of three independent experiments. Scale bars = 750 μm. Images were acquired on an EVOS FL digital inverted microscope (Thermo Fisher Scientific) using a 40× phase‐contrast objective with DAPI, FITC, and TRITC filter sets; exposure times were held constant across groups (auto‐exposure off). Acquisition and merges were performed in the EVOS software (version 1.4.1031.622). Only uniform, linear adjustments (global brightness/contrast) were applied equally to all images; no selective enhancement, smoothing, or feature editing was performed. Images represent post‐treatment conditions at 24 h (controls as indicated). Images were evaluated on a calibrated display under consistent ambient lighting. No arrows/labels were required because apoptotic/necrotic controls show diffuse, full‐field signals that are visually distinct from the absence of staining in experimental and untreated groups.

Although BSN reduced DPSC metabolic activity, cytotoxicity may still occur through apoptosis. To assess this, Annexin V/PI staining was used to distinguish early apoptosis from necrosis. Annexin V binds to phosphatidylserine exposed on the outer leaflet of the plasma membrane during early apoptosis (Shlomovitz et al. 2019), while PI penetrates and stains the nuclei of membrane‐compromised necrotic cells. Cells treated with methanol (positive control) showed normal nuclei staining (Figure 2A) and intense Annexin V staining (green), indicating apoptosis (Figure 2B), and strong PI nuclear staining (red) (Figure 2C). Merged images confirmed the cells had undergone necrosis (Figure 2D). In contrast, unstimulated control cells showed normal nuclei staining (Figure 2E) and an absence of Annexin V (Figure 2F) and PI staining (Figure 2G). Merged images indicated that there was no evidence of apoptosis or necrosis (Figure 2H). Likewise, BSN‐treated cells showed normal nuclei staining (Figure 2I) and an absence of Annexin V (Figure 2J) and PI staining (Figure 2K). Merged images indicated that there was no evidence of apoptosis or necrosis (Figure 2L).

Taken together, these results show that while BSN reduced metabolic activity and proliferation, it did not induce apoptotic or necrotic cell death in DPSCs.

### 3.2 Transcriptomic Profiling Reveals Dynamic Time‐Dependent Gene Expression Changes Following Exposure of Dental Pulp Stem Cells to Supernatant From a Four Species Interkingdom Endodontic Biofilm

Whole transcriptomic sequencing was next conducted to comprehensively evaluate the effect of BSN exposure on DPSCs. All sequenced samples met quality control standards with > 96% alignment to the human reference genome (data not shown). Principal component analysis (PCA) revealed a clear separation between groups, with the primary variance (PC1, 53.39%) driven by the treatment condition and the secondary variance (PC2, 14%) influenced by the duration of exposure (Figure S1B).

**Figure 3:**
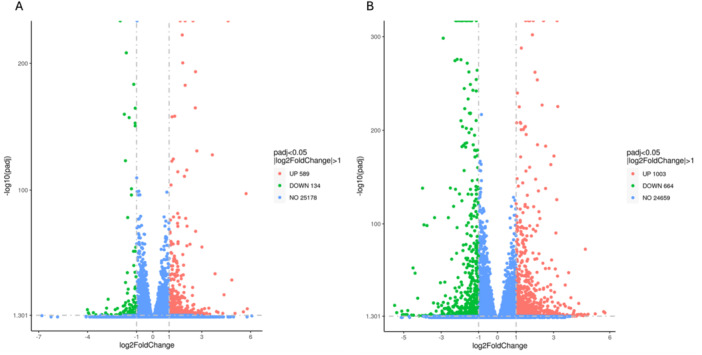
Differential gene expression in DPSCs following exposure to biofilm supernatant (BSN). Volcano plots showing the distribution of differentially expressed genes (DEGs) in DPSCs treated with BSN for 4 h (A) and 24 h (B) compared to untreated controls. Each point represents a gene. The x‐axis shows the log_2_ fold change, and the y‐axis shows –log_10_ (*p*adj), where 1.3 corresponds to *p*adj < 0.05. Red and green dots indicate significantly upregulated and downregulated genes (log_2_FC > 1, *p*adj < 0.05), respectively; blue dots indicate genes not significantly changed. Data are representative of four independent experiments.

**Figure 4:**
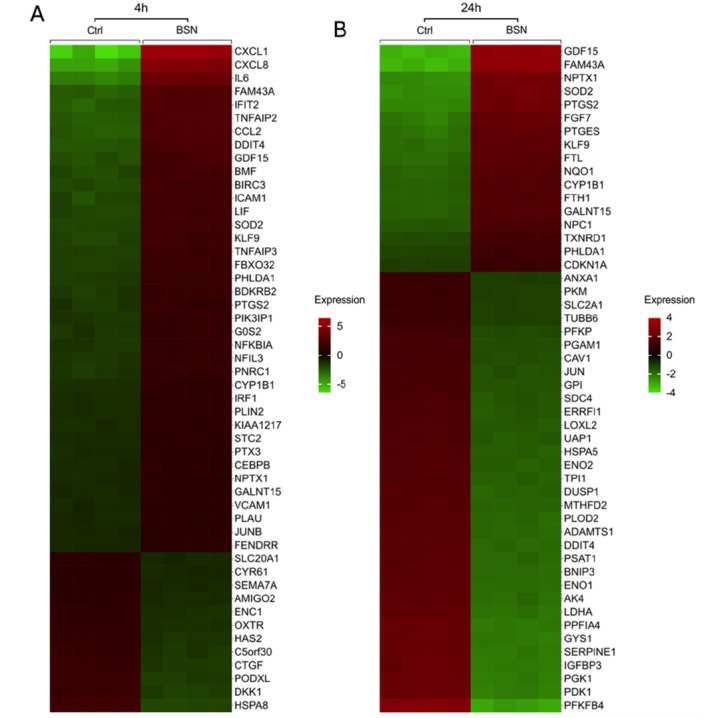
Heatmaps of top differentially expressed genes in DPSCs following biofilm supernatant (BSN) exposure. (A) Heatmap of the top 50 DEGs at 4 h. (B) Heatmap of the top 50 DEGs at 24 h. Expression values are based on normalized log_2_ fold change, where red indicates higher expression and green indicates lower expression relative to the control. Each row represents a gene; each column represents a biological replicate. Gene names are listed on the right. Data are representative of four independent experiments.

Analysis of differentially expressed genes (DEGs) demonstrated that BSN stimulation caused a substantial transcriptional response. After 4 h, 589 genes were significantly upregulated and 134 downregulated (Figure 3A), whereas at 24 h, 1003 genes were upregulated and 664 downregulated (Figure 3B). Heatmap analysis of the top 50 DEGs (ranked by *p*adj value) at each time point further illustrated the temporal dynamics of this response (Figure 4). At 4 h (Figure 4A), gene expression changes were dominated by upregulation of pro‐inflammatory and stress‐related genes, for example: *CXCL1*, *CXCL8*, *IL6*, *IFIT2*, *TNFAIP2,* and CCL2. By 24 h (Figure 4B), the expression profile shifted to include genes involved in redox balance, metabolism, and cellular signaling, for example: *SOD2*, *GDF15*, *CYP1B1*, and *NQO1*, suggesting a coordinated transition from acute immune activation toward regulatory and metabolic adaptation.

### 3.3 Supernatant From a Four Species Interkingdom Endodontic Biofilm Triggers Early Immune Activation Followed by Late Metabolic Adaptation

To explore the molecular pathways in DPSCs activated by BSN exposure, gene ontology (GO) enrichment analysis was performed on RNA‐seq datasets at 4 and 24 h post‐treatment.

**Figure 5:**
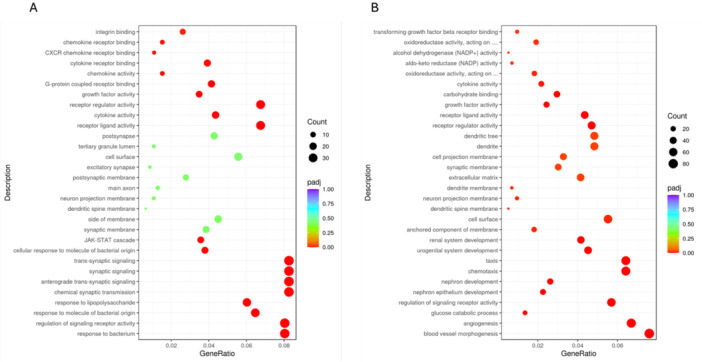
**Gene ontology (GO) enrichment analysis of differentially expressed genes in DPSCs following biofilm supernatant (BSN) exposure.** (A) GO enrichment at 4 h post‐exposure. (B) GO enrichment at 24 h post‐exposure. The top 10 significantly enriched terms are shown for each GO category: molecular function (MF), cellular component (CC), and biological process (BP). Dot size represents gene count; color scale indicates adjusted *p*‐value (*p*adj), with red denoting the most significant terms. GeneRatio reflects the proportion of genes enriched in each category relative to the total number of annotated genes.

At 4 h, the transcriptional profile showed strong enrichment in immune‐related biological processes (Figure 5A). The most significantly upregulated pathways included response to bacterium, response to molecules of bacterial origin, response to lipopolysaccharide, and the JAK‐STAT cascade (*p*adj < 0.001). Key upregulated genes driving these enrichments included *CXCL8*, *IL6*, *CCL2*, *NFKBIA*, and *ICAM1*, indicating a robust early innate immune response to BSN (Table S3). In contrast, downregulated pathways at 4 h were related to neuronal development and differentiation, such as positive regulation of neuron differentiation and neurogenesis, suggesting early suppression of developmental processes (*p*adj < 1e−4) (Table S3).

By 24 h, the transcriptional landscape had shifted. Enrichments were observed in pathways associated with angiogenesis, blood vessel morphogenesis, and signaling receptor regulation, reflecting a transition toward tissue remodeling and adaptation (*p*adj *P* < 1e−6) (Figure 5B and Table S3). Concurrently, glycolysis‐related pathways including NADH regeneration, canonical glycolysis, and glucose catabolic process were significantly downregulated, indicating suppression of metabolic activity and cellular energy output (*p*adj < 1e−10) (Table S3).

Collectively, these findings suggest that BSN induces a time‐dependent transcriptional program in DPSCs characterized by an early immune activation phase followed by a later metabolic and structural adaptation phase.

**Figure 6:**
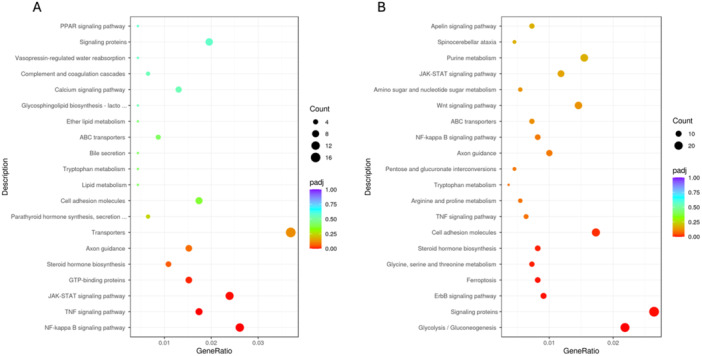
KEGG pathway enrichment analysis of differentially expressed genes in DPSCs following biofilm supernatant (BSN) exposure. (A) KEGG enrichment at 4 h post‐exposure. (B) KEGG enrichment at 24 h postexposure. Dot plots show the top significantly enriched pathways based on adjusted *p*‐values. Dot size reflects the number of enriched genes; color scale represents *p*adj, with red indicating higher significance. GeneRatio indicates the proportion of differentially expressed genes within each pathway relative to the total number of genes annotated to that pathway.

To complement the gene ontology results, KEGG pathway enrichment analysis was performed on differentially expressed genes at both 4 and 24 h post BSN exposure. At 4 h, BSN‐treated DPSCs showed strong activation of inflammatory and immune‐related pathways, including the NF‐κB signaling pathway (*p*adj = 1.2e−6), TNF signaling (*p*adj = 1.9e−4), and JAK‐STAT signaling (*p*adj = 0.003) pathways (Figure 6A and Table S4). These enrichments were driven by key regulators such as *CXCL8*, *IL6*, *NFKBIA*, *STAT2*, and *NOD2*, supporting a rapid immunotranscriptional response. Pathway enrichment was largely due to upregulated genes; no KEGG terms were significantly enriched among downregulated genes at this early timepoint.

By 24 h, KEGG enrichment highlighted a marked suppression of glycolysis/gluconeogenesis (*p*adj = 1.99e−6), consistent with broad metabolic inhibition (Figure 6B and Table S4). Additional pathway changes included activation of ErbB signaling (*p*adj = 0.0045) and stress responses such as ferroptosis (*p*adj = 0.0051). Intermediary metabolism was also affected, involving glycine/serine/threonine metabolism (*p*adj = 0.0097) and upregulation of steroid hormone biosynthesis (*p*adj = 0.0097). Finally, cell adhesion molecules were downregulated (*p*adj = 0.0176), suggesting altered cell–cell and cell–matrix interactions under BSN exposure. Together, these changes align with the reduced metabolic activity and proliferation observed in Figure 1, while the upregulation of ferroptosis and steroid biosynthesis points to stress adaptation and altered metabolic signaling.

Together, KEGG pathway analysis reinforces the biphasic nature of the BSN‐induced response, with an initial immune activation followed by a late‐phase shift in metabolic and signaling pathway enrichment.

### 3.4 Validation of IL8 and IL6 Upregulation at the Transcript and Protein Level

**Figure 7:**
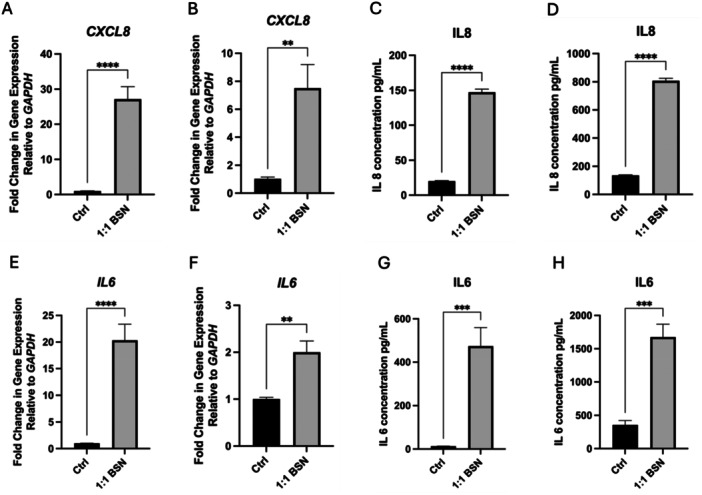
IL8 and IL6 expression by DPSCs following biofilm supernatant (BSN) exposure. Quantitative PCR analysis of *CXCL8* expression after 4 h (A) and 24 h (B) exposure to 1:1 BSN, normalized to *GAPDH*. ELISA measurements of IL8 protein levels in culture supernatants after 4 h (C) and 24 h (D) of BSN exposure. Quantitative PCR analysis of IL6 expression after 4 h (E) and 24 h (F) exposure to 1:1 BSN, normalized to *GAPDH*. ELISA measurements of IL6 protein levels in culture supernatants after 4 h (G) and 24 h (H) of BSN exposure. Both IL8 and IL6 showed significant upregulation at transcript and protein levels in response to BSN treatment at each time point compared to untreated controls. Data are presented as mean ± SEM from three independent experiments. **p < 0.01, ***p < 0.001, and ****p < 0.0001.

To validate the RNA‐seq findings and correlate gene expression with protein output, IL8 (CXCL8) and IL6 transcript and protein levels were quantified in the same samples used for sequencing. At 4 h, BSN stimulation induced a ~ 27‐fold increase in IL8 mRNA relative to GAPDH (*p* < 0.0001), with a ~ 7‐fold increase at 24 h (*p* < 0.01) (Figure 7A,B). IL‐8 protein levels in the bathing supernatant also significantly increased at 4 and 24 h (both *p* < 0.0001) (Figure 7C,D). Similarly, IL6 mRNA expression increased ~20‐fold at 4 h (*p* < 0.0001) and ~2‐fold at 24 h (*p* < 0.01) (Figure 7E,F). IL‐6 protein levels in the bathing supernatant also significantly increased at 4 and 24 h (both *p* < 0.0001) (Figure 7G,H). These findings agree with the transcriptional changes observed in the RNA‐seq analysis.

## 4 Discussion

This study provides direct evidence that exposure to supernatant derived from a polymicrobial interkingdom deep caries‐like biofilm elicits a potent and time‐dependent transcriptional response in human DPSCs, marked by early immune activation followed by later metabolic remodeling. These findings highlight the complex interplay between DPSCs and biofilm‐derived products in deep caries and carious pulp exposures and emphasize the relevance of host–biofilm interactions in inflamed, still‐vital, dental pulp and their importance to the outcome of VPT.

Regenerative strategies in endodontics span two distinct clinical settings. For necrotic pulp, regenerative endodontic procedures primarily rely on stem and progenitor cell sources present at the root apex, including cells from the apical papilla and adjacent apical tissues (Scelza et al. 2021; Saoud et al. 2016). In contrast, VPT targets inflamed yet still vital pulp, where DPSCs remain present and functionally relevant. The present study focuses on this latter context, in which preserving DPSC function under inflammatory pressure is central to clinical success.

The early induced expression of inflammatory mediators such as IL8, IL6, and TNFAIP2 at 4 h by DPSCs is consistent with innate immune sensing of microbial products in deep carious lesions and carious pulp exposures, where resident pulp cells rapidly release cytokines that drive localized pulp inflammation. Clinical microbiome studies of advanced caries with pulpitis similarly link the presence of Streptococcus, Fusobacterium, and Porphyromonas species with throbbing pain, percussion sensitivity, and other symptomatic presentations (Rôças et al. 2015), reinforcing the link between microbial activity and cytokine‐driven pulp symptoms. Indeed, rapid cytokine release has also been implicated in the development of periapical inflammatory lesions and associated symptoms, as reported in clinical and experimental studies (Stashenko et al. 1998). Enrichment of JAK‐STAT and NF‐κB signaling pathways further supports the view that DPSCs contribute actively to immune regulation in infected pulp tissue, a role increasingly recognized in endodontic immunobiology (Chen et al. 2024).

The four‐species endodontic biofilm model used in this study was selected due to its clinical relevance to biofilm reported to be present at the caries–pulp interface. *S. gordonii* is an abundant colonizer in advanced dentinal caries; *F. nucleatum* and *P. gingivalis* have been detected in deep lesions and carious exposures; and *Candida albicans* has been recovered from deep proximal caries (Rôças et al. 2015, 2016; Neves et al. 2017; Kuzmanović Radman et al. 2019; Kirilova et al. 2019). Furthermore, lipopolysaccharide (LPS) from *Fusobacterium nucleatum* and *Porphyromonas gingivalis* can provoke robust IL‐1β and TNF‐α responses from macrophages, underscoring the pro‐inflammatory potential of these species (Martinho et al. 2016). In inflamed, yet vital pulp, it is rational to assume that diffusible products from such communities of organisms can modulate DPSC behavior and therefore play a role in the success of VPT.

Although BSN reduced DPSC metabolic activity, it did not trigger apoptotic or necrotic cell death. This suggests a stress‐adaptive state rather than irreversible injury, which is critical when considering the regenerative capacity of DPSCs within infected environments. Indeed, by 24 h, the transcriptional signature had shifted toward redox balance, angiogenesis, and suppressed energy metabolism, including ferroptosis and glycolysis inhibition. These transitions are aligned with a sustained pulp inflammatory state in deep caries, where repair signals and immune modulation coexist, and suggest that DPSCs maintain their regenerative potential under inflammatory pressure. However, the interplay between host cells and complex multispecies biofilm in vivo needs to be taken into consideration. Indeed, a limitation of this study is the use of a simplistic two‐dimensional in vitro system. The absence of a 3D pulp‐like extracellular matrix and the lack of immune and vascular components restrict the ability to fully replicate the structural complexity, nutrient gradients, and biomechanical cues present in vivo. Simplified in vitro biofilm models also often fail to capture the spatial organization, chemical heterogeneity, and interkingdom interactions seen in clinical infections, which can significantly alter microbial virulence and host cell responses (Brown et al. 2019). Furthermore, in vivo, DPSCs are influenced by dynamic crosstalk with immune cells, endothelial cells, and resident microbiota, and these interactions, along with fluctuating oxygen levels and shear forces, may substantially modify both their metabolic behavior and that of the associated biofilm. Consequently, the translational relevance of these findings depends on future validation in more physiologically relevant 3D or in vivo models.

The findings from this study reinforce the notion that soluble biofilm‐derived factors can modulate host stem cell behavior without the need for direct microbial contact. In deep dentine adjacent to an inflamed vital pulp, diffusible biofilm products can traverse dentinal tubules and alter DPSC behavior even without bacterial invasion of the pulp tissue. The early immune activation followed by metabolic down‐tuning observed in this study is biologically consistent with pulp inflammation in deep caries, providing a mechanistic rationale for detoxification and biocompatible capping protocols that protect DPSC function prior to tissue decompensation. Given that virulence factors such as short‐chain fatty acids (SCFAs), proteases, and outer membrane vesicles from *P. gingivalis*, *F. nucleatum*, and *C. albicans* can diffuse through dentine (Yoo et al. 2020; Du et al. 2022), the present data provide molecular insight into how these diffusible products may modulate regenerative potential even after bacterial clearance.

A limitation of the present study is the absence of detailed chemical or molecular characterization of the biofilm supernatant. While the interkingdom multispecies biofilm model employed here is known to generate a broad spectrum of virulence‐associated metabolites and antigens relevant to endodontic infections, the specific components responsible for the observed biphasic immune–metabolic response in DPSCs were not individually identified. Future studies incorporating targeted biochemical or omics‐based analyses will be necessary to further elucidate the molecular drivers of these cellular responses. Another limitation of the present study is the absence of direct functional assays assessing DPSCs differentiation and migration. Although these properties are essential for pulp repair and regeneration, the current work focused on early cellular viability and immune–metabolic transcriptional responses to biofilm‐derived soluble factors. These early responses are known to critically shape downstream stem cell fate decisions, including differentiation and migratory behavior. Future studies incorporating targeted differentiation and migration assays will be necessary to directly link biofilm‐derived stimuli to these regenerative outcomes.

Interleukin‐8 (IL‐8; CXCL8) was demonstrated to be significantly highly expressed in response to BSN. It has previously been detected in inflamed dental pulp and periapical tissues (Hirsch et al. 2017; Galler et al. 2021), and its persistent elevation here reinforces its role as both a chemokine and a potential modulator of stem cell fate under inflammatory pressure. Similarly, IL6 expression was markedly increased in response to BSN, consistent with its known role in pulpal inflammation and immune signaling. Beyond its pro‐inflammatory function, IL6 has been demonstrated to influence mesenchymal stem cell behavior by affecting proliferation, differentiation, and migration, making it highly relevant in the context of DPSC‐mediated repair (Xie et al. 2018; Bastidas‐Coral et al. 2016). Modulating these cytokine pathways may therefore represent a therapeutic entry point to balance immune regulation and regeneration.

Together, these findings support a mechanistic model in which early immune activation is followed by metabolic adaptation, with direct implications for VPT and the design of regenerative and immunomodulatory biomaterials. A deeper understanding of the biological effects of diffusible products from complex biofilms could therefore inform timing, material choice, and detoxification strategies aimed at preserving pulp vitality in deep caries. In addition, it could identify novel adjunctive treatment strategies aimed at controlling pulpal inflammation whilst preserving the regenerative potential of vital tissues.

## 5 Conclusion

This study demonstrates that soluble factors secreted by a clinically relevant four‐species endodontic biofilm can elicit a time‐dependent immunometabolic transcriptional response in DPSCs. The early induction of pro‐inflammatory mediators, followed by a shift toward metabolic suppression and tissue remodeling pathways, reflects pulpal responses in deep caries and carious exposures. These data support the concept that diffusible biofilm byproducts can modulate DPSC function, reinforcing the need for detoxification and biologically sensitive VPT protocols that protect the inflamed yet vital pulp. These findings highlight the capacity of biofilm‐derived by‐products to modulate the regenerative function of DPSCs even in the absence of direct microbial contact. Future studies incorporating three‐dimensional or in vivo models are warranted to validate these observations and guide the development of therapeutic strategies aimed at preserving pulp vitality in the inflamed yet vital pulp.

## Author Contributions

**Sumaya Abusrewil:** conceptualization, experimental design, investigation, data analysis, data interpretation, writing – original draft. **Sumaya Abusrewil**, **Othman Baradwan**, and **Om Alkhir Alshanta:** experimental design, investigation, data collection, validation. **Ryan Kean** and **Jason L. Brown:** supervision, data analysis, data interpretation. **William McLean:** writing – review and editing. **Christopher J. Nile:** supervision, conceptualization, experimental design, data analysis, data interpretation, writing – review and editing.

## Ethics Statement

Ethical approval was not required. Human dental pulp stem cells (DPSCs) were commercially sourced from Lonza (UK) and used in vitro; no human participants or animals were directly involved.

## Consent

Patient consent was not required, as no human participants were involved in this study.

## Conflicts of Interest

The authors declare no conflicts of interest.

## Supporting information


Supporting File 1

Supporting File 2

Supporting File 3

## Data Availability

The RNA‐seq data supporting this study have been deposited in NCBI's Gene Expression Omnibus and are accessible through GEO Series accession number GSE308847 (https://www.ncbi.nlm.nih.gov/geo/query/acc.cgi?acc=GSE308847). All remaining raw data and materials are available upon reasonable request from the corresponding author.
