# Supplementary material for: Interkingdom Endodontic Biofilm Supernatant Induces a Biphasic Inflammatory and Metabolic Transcriptional Response in Dental Pulp Stem Cells In Vitro
Source: Clin Exp Dent Res. 2026 Jul 19;12(4):e70417. doi: 10.1002/cre2.70417 (PMC13380816; doi:10.1002/cre2.70417)
Supplement: Supplementary file 1 — Supporting File 1 [file CRE2-12-e70417-s003.docx]

**Supplementary Figure 1**

**
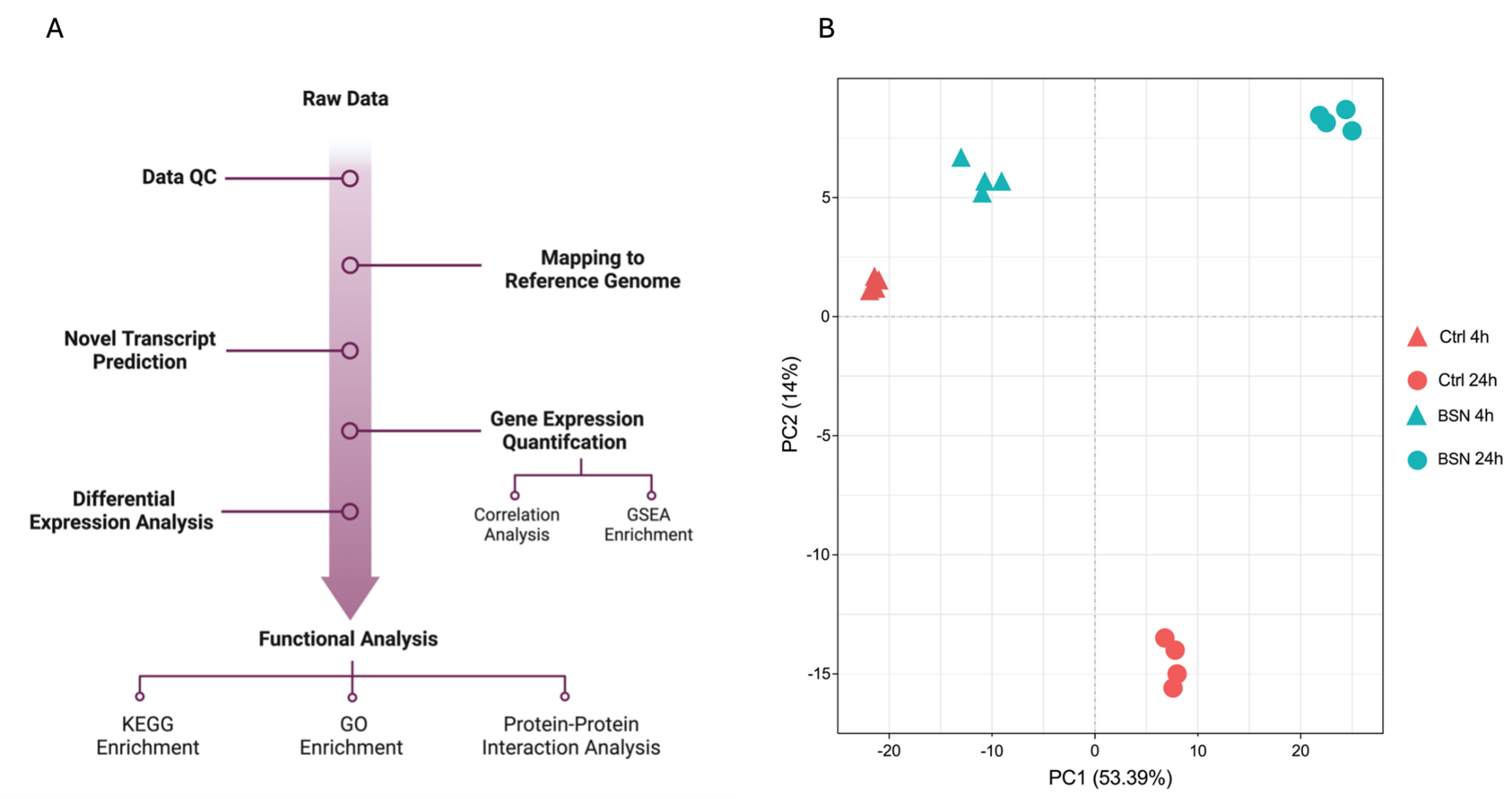
**

**Supplementary Figure 1. RNA-seq data processing and sample clustering.**

**(A)** Bioinformatic analysis pipeline. The schematic diagram shows the bioinformatics workflow for RNA sequencing data analysis. Raw sequences underwent quality control to ensure high-fidelity reads, followed by alignment to the human reference genome. Gene expression was quantified, and differentially expressed genes were identified. Functional enrichment analyses were performed using GO, KEGG, and protein–protein interaction databases. **(B)** Principal component analysis (PCA). PCA plot displaying clustering of samples based on variance in gene expression following DPSC exposure to 1:1 BSN for 4 h and 24 h. PC1 (x-axis) explains 53.39% of the total variance, while PC2 (y-axis) accounts for 14%. Distinct separation between treated and control groups confirms the impact of BSN on global transcriptional profiles.

**Supplementary Table1. Primer sequences used for qPCR.**

| Gene | Forward primer 5′−3′ | Reverse primer 5′−3′ | Reference |
| --- | --- | --- | --- |
| *IL8* | CAGAGACAGCAGAGCACACAA | TTAGCACTCCTTGGCAAAAC | (40) |
| *IL6* | GGTACATCCTCGACGGCATCT | GTGCCTCTTTGCTGCTTTCAC | (41) |
| *GAPDH* | CAAGGCTGAGAACGGGAAG | GGTGGTGAAGACGCCAGT | (40) |

**Supplementary Table2.**

| Assay | BSN dilution | Mean | Std. Error of Mean | Mean diff vs Ctrl | 95% CI | p-value |
| --- | --- | --- | --- | --- | --- | --- |
| MTT | 1:1 | 0.23 | 0.02 | 0.23 | 0.1479 to 0.3088 | <0.0001 |
|  | 1:2 | 0.35 | 0.02 | 0.11 | 0.02955 to 0.1905 | 0.0039 |
|  | 1:3 | 0.43 | 0.02 | 0.025 | -0.05583 to 0.1051 | 0.8909 |
|  | 1:4 | 0.42 | 0.03 | 0.032 | -0.04872 to 0.1122 | 0.7570 |
| CCK-8 | 1:1 | 1.20 | 0.02 | 0.59 | 0.3792 to 0.7971 | <0.0001 |
|  | 1:2 | 1.59 | 0.059 | 0.20 | 0.01080 to 0.4071 | 0.0685 |
|  | 1:3 | 1.70 | 0.039 | 0.09 | -0.1160 to 0.3020 | 0.6873 |
|  | 1:4 | 1.83 | 0.043 | -0.04 | -0.2505 to 0.1675 | 0.9873 |
| LDH | 1:1 | 0.00 | 0.00 | 2.92 | N/A | N/A |

**Supplementary Table3**

**Gene Ontology (GO) enrichment analysis of DPSCs following exposure to 1:1 BSN.**

The multi-sheet supplementary file presents significantly enriched GO pathways identified at 4 and 24 hours post-treatment. For each timepoint, results are organized into three sheets: an overview of all enriched pathways, followed by separate lists of upregulated and downregulated gene sets. Sheets 1–3 correspond to the 4-hour dataset, and Sheets 4–6 to the 24-hour dataset. Enrichment was based on differentially expressed genes with adjusted p-values (padj ≤ 0.05).

**Supplementary Table4. KEGG pathway enrichment analysis of DPSCs following exposure to 1:1 BSN.**

The multi-sheet supplementary file presents significantly enriched KEGG pathways identified at 4 and 24 hours post-treatment. For each timepoint, results are organized into three sheets: an overview of all enriched pathways, followed by separate lists of upregulated and downregulated gene sets. Sheets 1–3 correspond to the 4-hour dataset, and Sheets 4–6 to the 24-hour dataset. Enrichment was based on differentially expressed genes with adjusted p-values (padj ≤ 0.05).
